# Optimal Pricing and Power Allocation for Collaborative Jamming with Full Channel Knowledge in Wireless Sensor Networks

**DOI:** 10.3390/s17112697

**Published:** 2017-11-22

**Authors:** Dae-Kyo Jeong, Insook Kim, Dongwoo Kim

**Affiliations:** 1 Department of Electronics and Communication Engineering, Hanyang University, Ansan 15588, Korea; dkjeong@wnl.hanyang.ac.kr; 2 Division of Electrical Engineering, Hanyang University, Ansan 15588, Korea; kimins@hanyang.ac.kr

**Keywords:** optimal pricing, secure capacity, power allocation, Stackelberg game, distributed pricing

## Abstract

This paper presents a price-searching model in which a source node (Alice) seeks friendly jammers that prevent eavesdroppers (Eves) from snooping legitimate communications by generating interference or noise. Unlike existing models, the distributed jammers also have data to send to their respective destinations and are allowed to access Alice’s channel if it can transmit sufficient jamming power, which is referred to as collaborative jamming in this paper. For the power used to deliver its own signal, the jammer should pay Alice. The price of the jammers’ signal power is set by Alice and provides a tradeoff between the signal and the jamming power. This paper presents, in closed-form, an optimal price that maximizes Alice’s benefit and the corresponding optimal power allocation from a jammers’ perspective by assuming that the network-wide channel knowledge is shared by Alice and jammers. For a multiple-jammer scenario where Alice hardly has the channel knowledge, this paper provides a distributed and interactive price-searching procedure that geometrically converges to an optimal price and shows that Alice by a greedy selection policy achieves certain diversity gain, which increases log-linearly as the number of (potential) jammers grows. Various numerical examples are presented to illustrate the behavior of the proposed model.

## 1. Introduction

Due to the inherent broadcast nature of the wireless medium, eavesdropping is one of major threats in wireless network security. Especially, wireless sensor networks, where tiny sensing devices are transmitting information data through radio links, are vulnerable to eavesdroppers (Eves). Physical layer security emerges as an effective means of securing wireless communications against eavesdropping by exploiting the physical characteristics of wireless channels. In the presence of an Eve, a so-called secrecy capacity is given in [1,2] as the difference between the channel capacity from a source (referred to as Alice) to destination (referred to as Bob) and that from the source to Eve.

In tremendous research efforts in enhancing physical layer security (for example, listed in [3]), jamming has been widely accepted as an attractive way, which prevents Eves from snooping legitimate communications by generating friendly interference or noise. In [4], the coverage of friendly jamming is evaluated, and the secrecy outage probability (SOP) is characterized for various levels of channel state information. In [5], optimal power allocation between multiple friendly jammers that cooperatively jam multiple Eves is investigated in order to maximize the secrecy capacity, and SOP is also analyzed. Jamming signals are sometimes transmitted by intermediate relay nodes to secure the source signals, which is so-called cooperative jamming [6,7]. In [8], multiple intermediate nodes are designated as either relays or jammers, for which optimal relay and jammer selection is investigated. In [9], the cooperative jammers harvest energy transmitted by a source and use it to generate artificial noise to jam the Eves cognitive Internet-of-Things networks, in which an auction framework that formulates the jammer selection and the power allocation together is also provided. In an amplify-and-forward relay network, the destination can work as a friendly jammer by generating artificial noise in the first phase to prevent the possibly untrusted relay from decoding the signal [10].

Though there have been plenty of works on investigating the secrecy performance of friendly or cooperative jamming, to the best of our knowledge, why and how much the jammers waste their valuable power or time for Alice is not well modeled in the literature. In [11], a game-theoretic model is established for this purpose, where Alice pays the jammer for the jamming power to interfere with Eve, therefore achieving more secrecy capacity, and the relationship between the jamming power and the price is investigated.

In this paper, we pay attention to the great burden that Alice should suffer in the previous model, though it is a legitimate user having a valuable right to access certain bandwidth. Our model relieves its burden by allowing the jammer to send its own data as a secondary legitimate user and letting Alice charge for the power used to deliver the jammer’s signal, which is referred to as collaborative jamming in this paper. The proposed collaborative jamming is more efficient than the previous jamming techniques in the sense that the jamming power is not just wasted, but used to send information data. To maintain the communication quality of Alice, the collaboration is allowed only if it does not degrade a predetermined level of secrecy capacity.

With the collaboration, we provide an optimal price issued by Alice and an optimal power allocation by the jammer corresponding to the price, where Alice is often called a price-leader and Jack is a price-follower in Stackelberg games. In order to provide the optimal solutions, we assume that legitimate sensors have perfect and full knowledge of Eve’s channel in their vicinity. We further investigate two scenarios for the collaboration: Bob and the jammer share common artificial signals (CASs) that are used for jamming signals so that Bob could (partly) cancel the jamming interference from the received signals; Additionally, Bob and the jammer have no CASs, and thus, the whole power from the jammer is treated as interference by Bob. We further extend the model with multiple jammers, in which Alice selects the most beneficial jammer that would provide the greatest payment. With such a greedy-selection policy, it is shown that the benefit taken by Alice increases as the number of potential jammers increases. Moreover, the probability (in asymptotic environments) that Alice’s benefit drops below a certain predefined threshold is shown to decrease geometrically as the number of jammers grows. If multiple jammers exist, it is hard for Alice to know the network-wide channel information for jammers and/or for Eves. By assuming that only the jammers, but Alice, know the relevant channel information, an interactive and iterative price-searching procedure is provided. The procedure converges geometrically to an optimal price. With numerical investigation, we show that the convergence speed is sufficiently fast with the moderate number of jammers. Numerical examples are also provided for illustrating the proposed optimal pricing and power allocation for a single jammer and multiple jammers, respectively. The result especially show that about a 6–28% increase in Alice’s benefit is achieved by allowing eight jammers to participate in the price-searching procedure.

The contributions of this paper can be summarized as follows:

- provides a new price-searching model that explains an adequate price level of the transmitting power used to deliver secondary traffic by friendly jammers; who also contribute to protecting the secure transmission of Alice,
- presents, in closed-form, an optimal price that maximizes Alice’s benefit and the corresponding optimal power allocation from a jammers’ perspective;
- provides a distributed and interactive price-searching procedure that can be applied for multiple-jammer scenarios and geometrically converges to an optimal price; and
- shows that Alice by a greedy selection policy achieves a certain diversity gain, which increases log-linearly as the number of (potential) jammers grows.

The remainder of the paper is organized as follows. The system model that includes the collaboration condition and signal representations in the collaboration is provided in Section 1, In Section 2, optimal pricing and power allocation are provided for a single-jammer case. An optimal price is presented in closed-form for both scenarios: with and without CAS, respectively. Section 3 extends the result with a single jammer to a case of multiple jammers. When Alice selects the best jammer among potential jammers, it is shown that it enjoys a certain diversity gain that log-linearly increases as the number of jammers grows. In order to cope with a more practical problem in which Alice cannot gather the whole channel information in the network, a distributed and interactive collaboration search procedure (ICSP) is also provided in Section 3. The convergence of ICSP is numerically investigated in Section 4, in which a variety of other numerical examples is also discussed. Finally, conclusions are presented in Section 5. The main notations used in this paper are listed in Table 1.

## 2. System Model

### 2.1. Collaboration Model

**Figure 1:**
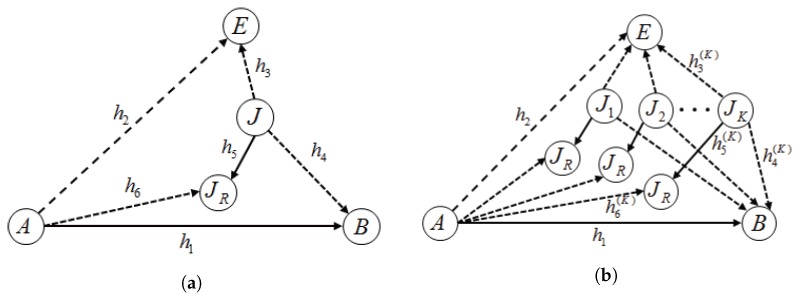
The system models: (**a**) single-jammer model; (**b**) multiple-jammer model.

We first consider a wireless network that consists of two source-destination pairs and an eavesdropper: (A,B), $\left(J,J_{R}\right)$ and *E*, respectively, as shown in Figure 1a. The system with multiple jammers (in Figure 1b) is investigated in Section 4. Alice (*A*) and Bob (*B*) are primary legitimate users where primary source Alice is allocated radio resources such as a unit time slot and transmit power $P_{A}$ to send its own data to Bob. On the other hand, secondary source Jack (*J*) has been partly allowed to access the time slot only if it does not degrade communication quality between the primary users by certain collaboration to be defined below. Eve (*E*) is another party that is eavesdropping the primary communication. We assume that Eve does not care about Jack’s messages and treats it as interference.

Jack seeks an opportunity to send signals to $J_{R}$ by jamming Eve to prevent it from eavesdropping Alice’s signal. Let $R_{AB}^{s}$ be a secrecy rate target desired by Alice. Alice allows Jack to access the time slot if Jack has sufficient jamming power by which $R_{AB}^{s}$ can be maintained and pays λ (dollars) per power unit that is used to send Jack’s own data. In summary, the collaboration between Alice and Jack could be established if:

- the collaboration would not have dropped the secrecy rate between the primary users below $R_{AB}^{s}$;
- Jack would pay λ to Alice for a unit power that it allocates to its own signal during the collaboration; and
- the benefit expected by the collaboration would be positive for both Alice and Jack.

We assume that Jack has transmit power $P_{J}$. For a given λ, Jack would maximize its utility of $P_{J}$ by deciding whether it joins the collaboration and how much power it will use to send its own data and pay the charge. Alice wants to attract Jack and maximize its revenue by setting an adequate price λ.

Alice is often called a price-leader and Jack is a price-follower in Stackelberg games. Our main interest lies in finding optimal λ that maximizes Alice’s revenue and Jack’s optimal power allocation strategies.

### 2.2. Signal Model for the Collaboration

Let $h_{1}$, $h_{2}$, $h_{3}$, $h_{4}$, $h_{5}$ and $h_{6}$ (shown in Figure 1) be the complex channel coefficients between *A*-*B*, *A*-*E*, *J*-*E*, *J*-*B*, *J*-$J_{R}$ and *A*-$J_{R}$ links, respectively. The channels are assumed to be static during the collaboration. Let $x_{1}$ and $x_{2}$ be complex symbol vectors for the message transmitted by Alice to Bob and Jack to $J_{R}$, respectively. If Alice and Jack (also $J_{R}$) have common artificial signals (CASs), Jack designates them as specific jamming signals denoted by $x_{J}$. Otherwise, $x_{J}$ is not used. When CAS exits, Jack transmits a superimposed signal $\sqrt{\alpha P_{J}}x_{2}+\sqrt{\left(1-\alpha \right)P_{J}}x_{J}$ where α(0≤α≤1) represents a power allocation factor between message signal $x_{2}$ and artificial signal $x_{J}$. If CAS does not exist, α=1 (it is also shown to be optimal shortly). Let $\eta_{\left\{\cdot \right\}}$ be noise power at the receivers. The signals received at Bob and $J_{R}$ are:

(1) $$\begin{array}{c} y_{B}=h_{1}\sqrt{P_{A}}x_{1}+h_{4}\left(\sqrt{\alpha P_{J}}x_{2}+\sqrt{\left(1-\alpha \right)P_{J}}x_{J}\right)+\eta_{B}, \end{array}$$

(2) $$\begin{array}{c} y_{J_{R}}=h_{5}\left(\sqrt{\alpha P_{J}}x_{2}+\sqrt{\left(1-\alpha \right)P_{J}}x_{J}\right)+h_{6}\sqrt{P_{A}}x_{1}+\eta_{J_{R}}, \end{array}$$

respectively. Eve also receives the signals simultaneously such that:

(3) $$y_{E}=h_{2}\sqrt{P_{A}}x_{1}+h_{3}\left(\sqrt{\alpha P_{J}}x_{2}+\sqrt{\left(1-\alpha \right)P_{J}}x_{J}\right)+\eta_{E}.$$

We assume that the transmitted symbols have zero mean and unit power. The complex additive Gaussian noises with zero mean and variance $\sigma^{2}$ are assumed for $\eta_{B},\eta_{J_{R}}$ and $\eta_{E}$, respectively. For notational brevity, let $\gamma_{i}=\frac{|h_{i}{|}^{2}}{\sigma^{2}}$ for all *i*. We assume that Alice and Jack know the relevant channel information perfectly.

## 3. Optimal Pricing and Power Allocation

### 3.1. When CAS Exists

#### 3.1.1. With Fixed $P_{J}$

When CAS exits, $x_{J}$ is known to Bob, as well as $J_{R}$ and can be canceled at the respective receivers. Thus, the channel capacities over *A*-*B* and *A*-*E* are given by:

(4) $$\begin{array}{ccc} C_{AB} & = & \log\left(1+\frac{\gamma_{1}P_{A}}{\gamma_{4}\alpha P_{J}+1}\right), \end{array}$$

(5) $$\begin{array}{ccc} C_{AE} & = & \log\left(1+\frac{\gamma_{2}P_{A}}{\gamma_{3}P_{J}+1}\right), \end{array}$$

respectively, where only $\alpha P_{J}$ is the interfering power from Jack at Bob since $\left(1-\alpha \right)P_{J}$ can be removed using an interference cancellation technique. Secrecy rate on *A*-*B* is then defined by [12]:

(6) $$C_{AB}^{s}≜\max\left\{C_{AB}-C_{AE},0\right\}.$$

The collaboration is then possible if:

(7) $$C_{AB}^{s}\geq R_{AB}^{s},$$

which is referred to as the collaboration allowance condition (CAC) in this paper.

Substituting (4) and (5) into (7), we obtain a collaboration-allowable power range:

(8) $$\alpha \leq \alpha_{0},$$

where:

(9) $$\alpha_{0}=\min\left[\frac{1}{\gamma_{4}P_{J}}\left(\frac{\gamma_{1}P_{A}}{2^{R_{AB}^{s}+C_{AE}}-1}-1\right),1\right].$$

$\alpha_{0}$ is a maximally-allowable fraction of Jack’s power to its own signals while keeping CAC. If $\alpha_{0}\leq 0$, CAC cannot be held with any positive power allocation, and then, the collaboration fails.

In the collaboration with Alice and Bob, Jack can achieve data rate:

(10) $$R_{J}=\log\left(1+\frac{\gamma_{5}\alpha P_{J}}{\gamma_{6}P_{A}+1}\right).$$

Let Jack have positive return $\omega_{J}$ (dollars) per the achieved data rate. The net revenue (utility) of Jack then can be modeled as:

(11) $$\begin{array}{cc} U_{J}^{}\left(\alpha ;\lambda \right) & =\;\omega_{J}R_{J}-\lambda \alpha P_{J}\\ & =\;\omega_{J}\log\left(1+\frac{\gamma_{5}\alpha P_{J}}{\gamma_{6}P_{A}+1}\right)-\lambda \alpha P_{J}. \end{array}$$

For given λ, $\omega_{J}$ and $P_{J}$, Jack finds an optimal power allocation $\alpha^{*}$ by solving:

(12) $$\max_{0\leq \alpha \leq \alpha_{0}}U_{J}^{}\left(\alpha ;\lambda \right).$$

By investigating the Karush–Kuhn–Tucker condition for (12), analogous to the method found in [13], we can have the following optimal power allocation.

**Lemma** **1** (Jack’s optimal power allocation). (13) $$\alpha^{*}\left(\lambda \right)=\left\{\begin{array}{cc} \alpha_{0}, & 0\leq \lambda \leq \lambda_{L}^{},\\ \frac{\omega_{J}}{\ln2}\frac{1}{\lambda P_{J}}-\frac{\gamma_{6}P_{A}+1}{\gamma_{5}P_{J}}, & \lambda_{L}\leq \lambda \leq \lambda_{U},\\ 0, & \lambda \geq \lambda_{U}. \end{array}\right.$$

*where:*

(14) $$\left\{\begin{array}{c} \lambda_{L}=\frac{\omega_{J}}{\ln2}\frac{\gamma_{5}}{\gamma_{5}\alpha_{0}P_{J}+\gamma_{6}P_{A}+1},\\ \lambda_{U}=\frac{\omega_{J}}{\ln2}\frac{\gamma_{5}}{\gamma_{6}P_{A}+1}. \end{array}\right.$$

For Alice and Bob, the payment by Jack can be regarded as compensation for the collaboration. If Jack optimizes its revenue with the power allocation in (13), the incentive for the primary users is then given by:

(15) $$U_{AB}\left(\lambda \right)=\lambda \alpha^{*}\left(\lambda \right)P_{J}=\left\{\begin{array}{cc} \lambda \alpha_{0}P_{J}, & 0\leq \lambda \leq \lambda_{L},\\ \frac{\omega_{J}}{\ln2}-\frac{\gamma_{6}P_{A}+1}{\gamma_{5}}\lambda , & \lambda_{L}\leq \lambda \leq \lambda_{U},\\ 0, & \lambda \geq \lambda_{U}. \end{array}\right.$$

If we assume that Alice further knows $\omega_{J}$ and $P_{J}$, Alice can maximize its incentive in (15). Obviously, $U_{AB}\left(\lambda \right)$ is maximized at $\lambda^{*}=\lambda_{L}$.

**Lemma** **2** (Optimal pricing by Alice). When Jack allocates transmit power to maximize its own revenue in (11), an optimal pricing policy by Alice is $\lambda^{*}=\lambda_{L}$, and the maximum incentive for Alice is $U_{AB}^{*}=\lambda_{L}\alpha_{0}P_{J}$.

#### 3.1.2. Optimal $P_{J}$

It is noted that Jack’s optimal power allocation is always $\alpha_{0}$ if Alice maximizes its incentive by disseminating an optimal price $\lambda_{L}$ and CAC holds. Keeping CAC, $\alpha_{0}\geq 0$ is equivalent to:

(16) $$P_{J}\geq \frac{1}{\gamma_{3}}\left(\frac{\gamma_{2}P_{A}}{\frac{\gamma_{1}P_{A}+1}{2^{R_{AB}^{s}}}-1}-1\right)≜P_{J}^{\left(C\right)}.$$

By substituting $\alpha_{0}$ into (11) and letting $P_{J0}≜\alpha_{0}P_{J}$, we have a new problem to determine $P_{J0}$ for Jack:

(17) $$\begin{array}{cc} \max_{P_{J0}} & U_{J}^{}\left(P_{J0};\lambda_{L}\right)=\omega_{J}\log\left(1+\frac{\gamma_{5}P_{J0}}{\gamma_{6}P_{A}+1}\right)-\lambda_{L}P_{J0}, \end{array}$$

for which an optimal solution is given by:

(18) $$P_{J0}^{\mathrm{opt}}=\frac{\omega_{J}}{\ln2}\frac{1}{\lambda_{L}}-\frac{\gamma_{6}P_{A}+1}{\gamma_{5}}.$$

Furthermore, let us denote the maximum available power at Jack by $P_{j}^{\max}$. An optimal $P_{J}$ is thus obtained by equating $\alpha_{0}P_{J}^{*}=P_{J0}^{\mathrm{opt}}$:

(19) $$P_{J}^{*}=\min\left[\max\left\{\frac{1}{\gamma_{3}}\left(\frac{\gamma_{2}P_{A}}{\frac{1}{2^{R_{AB}^{s}}}\left(\frac{\gamma_{1}P_{A}}{\gamma_{4}P_{J0}^{\mathrm{opt}}+1}+1\right)-1}-1\right),P_{J}^{\left(C\right)}\right\},P_{j}^{\max}\right].$$

### 3.2. When CAS Does Not Exist

In this case, Jack’s signals cannot be distinguished between jamming and data signals, and Alice assumes that the whole power from Jack is used to deliver Jack’s signal and hence sets α=1. For Jack, it is also optimal to set α=1, since it should pay $\lambda P_{J}$ if it uses power $P_{J}$. Let us denote the secrecy rate on *A*-*B* with α=1 by ${\tilde{C}}_{AB}^{s}$. Then, CAC ${\tilde{C}}_{AB}^{s}\geq R_{AB}^{s}$ gives a quadratic inequality on $P_{J}$ with interim constants a,b and *c* such that:

(20) $$\begin{array}{c} aP_{J}^{2}+bP_{J}+c\leq 0, \end{array}$$

where $a=\left(2^{R_{AB}^{s}}-1\right)\gamma_{3}\gamma_{4}$, $b=2^{R_{AB}^{s}}\gamma_{2}\gamma_{4}P_{A}-\gamma_{1}\gamma_{3}P_{A}+\left(2^{R_{AB}^{s}}-1\right)\left(\gamma_{3}+\gamma_{4}\right)$, and $c=\left(2^{R_{AB}^{s}}\gamma_{2}-\gamma_{1}\right)P_{A}+2^{R_{AB}^{s}}-1$. Since a>0, if $b^{2}-4ac<0$, CAC cannot hold for any real $P_{J}$; otherwise, letting $P_{J}^{\left(L\right)}$ and $P_{J}^{\left(U\right)}$ ($P_{J}^{\left(L\right)}\leq P_{J}^{\left(U\right)}$ where the equality holds if $b^{2}-4ac=0$ be the two real roots of (20), $P_{J}$ should be:

(21) $$P_{J}^{\left(L\right)}\leq P_{J}\leq P_{J}^{\left(U\right)}\;\;\mathrm{and}\;\;P_{J}\geq 0$$

in order to keep CAC. We assume in this paper that $P_{J}^{\left(U\right)}\geq 0$ to ensure the given problem is feasible.

For given λ, Jack is now solving:

(22) $$\max_{P_{J}}U_{J}^{}\left(P_{J};\lambda \right)=\omega_{J}\log\left(1+\frac{\gamma_{5}P_{J}}{\gamma_{6}P_{A}+1}\right)-\lambda P_{J},$$

under the constraint in (21). An optimal solution can be obtained by:

(23) $$P_{J}^{*}\left(\lambda \right)=\left\{\begin{array}{cc} P_{J}^{\left(U\right)}, & \lambda =0,\\ \max\left(P_{J}^{(L)+},\min\left(P_{J}^{\mathrm{opt}},P_{J}^{\left(U\right)},P_{j}^{\max}\right)\right), & \lambda >0 \end{array}\right.,$$

where $P_{J}^{(L)+}=\max\left(0,P_{J}^{\left(L\right)}\right)$ and:

(24) $$P_{J}^{\mathrm{opt}}=\frac{\omega_{J}}{\ln2}\frac{1}{\lambda }-\frac{\gamma_{6}P_{A}+1}{\gamma_{5}}.$$

It is noted that $P_{J}^{\mathrm{opt}}$ in (24) is an unconstrained optimal solution of (22) that is similar to (18).

For Alice, referring to (15), an optimal pricing policy is $\lambda_{L}$ with $\alpha_{0}=1$, which gives:

(25) $${\tilde{\lambda }}^{*}=\frac{\omega_{J}}{\ln2}\frac{\gamma_{5}}{\gamma_{5}P_{J}^{\left(V\right)}+\gamma_{6}P_{A}+1},$$

where $P_{J}^{\left(V\right)}=\min\left(P_{J}^{\left(U\right)},P_{j}^{\max}\right)$. Putting (25) into (24), we can have $P_{J}^{*}\left({\tilde{\lambda }}^{*}\right)=P_{J}^{\mathrm{opt}}=P_{J}^{\left(U\right)}$.

**Lemma** **3** (Optimal pricing and power allocation when CAS is not available). *When CAS is not available, an optimal pricing policy by Alice is ${\tilde{\lambda }}^{*}$, and an optimal transmit power by Jack is $P_{J}^{\left(V\right)}$ if feasible. The corresponding incentive for Alice is now $U_{AB}^{*}=\frac{\omega_{J}}{\ln2}\frac{\gamma_{5}P_{J}^{\left(V\right)}}{\gamma_{5}P_{J}^{\left(V\right)}+\gamma_{6}P_{A}+1}.$*

## 4. Extension to Multiple Jammers

### 4.1. Greedy Jammer Selection

Let $J_{C}$ and $J_{N}$ denote sets of jammers who have CASs or not, respectively, and let us assume $J_{C}\cup J_{N}\neq ⌀$. For jammer $j\in J_{C}$, we assume that each of them transmits at a maximum power level $P_{\max}^{\left(j\right)}$. Hereafter, superscript ${}^{\left(j\right)}$ is used to denote the respective jammers. For $j\in J_{N}$, we assume that each jammer has real roots for (20), and the greater one is denoted by $P_{U}^{\left(j\right)}\geq 0$; further assume that $P_{U}^{\left(j\right)}\leq P_{\max}^{\left(j\right)}$ without loss of generality.

When λ is given by Alice, jammers compute $\alpha^{(j)*}\left(\lambda \right)$ and $P^{(j)*}\left(\lambda \right)$ according to (13) and (23), respectively. Let the payment by each of the jammers be denoted by $U_{AB}^{\left(j\right)}\left(\lambda \right)$. Then:

(26) $$U_{AB}^{\left(j\right)}\left(\lambda \right)=\left\{\begin{array}{cc} \lambda \alpha^{(j)*}\left(\lambda \right)P_{\max}^{\left(j\right)}, & j\in J_{C}\\ \lambda P^{(j)*}, & j\in J_{N}. \end{array}\right.$$

Let us define:

(27) $$U_{AB}^{\max}\left(\lambda \right)≜\max\left\{U_{AB}^{\left(j\right)}\left(\lambda \right),\;\mathrm{for}\;\mathrm{all}\;j\in J_{C}\cup J_{N}\right\}.$$

Thus, optimal price λ for multiple jammers is given by:

(28) $$\lambda_{m}^{*}=\arg\max_{\lambda \geq 0}U_{AB}^{\max}\left(\lambda \right).$$

In (27), it can be seen that there exists at least one $j\in J_{C}\cup J_{N}$ such that $U_{AB}^{\max}\left(\lambda \right)=U_{AB}^{\left(j\right)}\left(\lambda \right)$ for given λ. Let us denote by *k* a jammer that provides $U_{AB}^{\max}\left(\lambda_{m}^{*}\right)=U_{AB}^{\left(k\right)}\left(\lambda_{m}^{*}\right)$. Thus, jammer *k* offers a maximum payment to Alice. Alice maximizes its utility by choosing jammer *k*, which is referred to as a greedy selection in this paper. Ties, if any, could be broken arbitrarily. Selected jammer *k* determines its optimal power allocation according to either (13) or (23).

### 4.2. Asymptotic Analysis

When there are *K* jammers, Alice can select one of them as a friendly jammer, which may provide a maximum payment. Let $U_{AB}^{\left(j\right)}$ be the maximum payment by jammer *j*, which is determined when an optimal price is offered by Alice. Then, Alice’s maximum utility with *K* cooperative jammers $U_{AB}^{\max}\left(K\right)$ can be:

(29) $$U_{AB}^{\max}\left(K\right)=\max\left\{U_{AB}^{\left(1\right)},U_{AB}^{\left(2\right)},\cdots ,U_{AB}^{\left(K\right)}\right\}.$$

$U_{AB}^{\max}\left(K\right)$ obviously increases as *K* increases, which means that Alice enjoys the benefit from the diversity of potential jammers with the greedy selection principle. To quantify this benefit, let us assume that Alice has a utility target μ and suffers from a utility outage when $U_{AB}^{\max}\left(K\right)\leq \mu$. The probability of utility outage is then defined by:

(30) $$\begin{array}{cc} P_{\mathrm{out}} & =\mathrm{Pr}\{U_{AB}^{\max}\left(K\right)\leq \mu \}\\ & =\mathrm{Pr}\{U_{AB}^{\left(1\right)}\leq \mu ,U_{AB}^{\left(2\right)}\leq \mu ,\cdots ,U_{AB}^{\left(K\right)}\leq \mu \}. \end{array}$$

We assume that $j\in J_{C}$ for brevity. It is easy to show that the following observation is analogously valid for $j\in J_{N}$.

As $P_{J}$ goes to infinity, $\lambda_{L}$ decreases and converges to:

(31) $$\frac{\omega_{J}}{\ln2}\frac{\gamma_{5}}{\gamma_{5}{\bar{P}}_{J}+\gamma_{6}P_{A}+1},$$

where ${\bar{P}}_{J}=\frac{1}{\gamma_{4}}\left(\frac{\gamma_{1}P_{A}}{2^{R_{AB}^{s}}-1}-1\right)$. Additionally, $U_{AB}^{*}$ also converges to:

(32) $$\frac{\omega_{J}}{\ln2}\frac{\gamma_{5}{\bar{P}}_{J}}{\gamma_{5}{\bar{P}}_{J}+\gamma_{6}P_{A}+1}.$$

When $P_{A}$ further goes to infinity, ${\bar{P}}_{J}\approx \gamma_{1}P_{A}/\left(\gamma_{4}\beta \right)$ where $\beta =2^{R_{AB}^{s}}-1$. An asymptotic expression of $U_{AB}^{\left(j\right)}$ is given by:

(33) $$\begin{array}{c} {\tilde{U}}_{AB}^{\left(j\right)}=\frac{\gamma_{1}\Omega^{\left(j\right)}}{\gamma_{1}\Omega^{\left(j\right)}+\beta }, \end{array}$$

where $\Omega^{\left(j\right)}=\gamma_{5}^{\left(j\right)}/\left(\gamma_{4}^{\left(j\right)}\gamma_{6}^{\left(j\right)}\right)$. Let:

(34) $$\Omega =\max\left\{\frac{\gamma_{5}^{\left(1\right)}}{\gamma_{4}^{\left(1\right)}\gamma_{6}^{\left(1\right)}},\frac{\gamma_{5}^{\left(2\right)}}{\gamma_{4}^{\left(2\right)}\gamma_{6}^{\left(2\right)}},\cdots ,\frac{\gamma_{5}^{\left(K\right)}}{\gamma_{4}^{\left(K\right)}\gamma_{6}^{\left(K\right)}}\right\}.$$

Then, the asymptotic outage probability becomes (if we assume that every jammer has the same $\omega_{J}$ for brevity):

(35) $$\begin{array}{ccc} {\tilde{P}}_{\mathrm{out}} & = & \mathrm{Pr}\left\{\frac{\gamma_{1}\Omega }{\gamma_{1}\Omega +\beta }\leq \frac{\mu \ln2}{\omega_{J}}\right\}\\ & = & \mathrm{Pr}\left\{\Omega \leq \frac{\beta }{\left(\frac{\omega_{J}}{\mu \ln2}-1\right)\gamma_{1}}\right\}\\ & = & \int_{0}^{\infty }F_{\Omega }\left(\frac{\beta \mu \ln2}{(\omega_{J}-\mu \ln2)x}\right)f_{\gamma_{1}}\left(x\right)dx, \end{array}$$

where $f_{\gamma_{1}}\left(x\right)$ and $F_{\Omega }\left(x\right)$ represent the PDF of $\gamma_{1}$ and the CDF of Ω, respectively.

We assume that $\gamma_{1}$ and $\gamma_{k}^{\left(j\right)}$ (k=4,5,6) have the exponential distribution with mean $m_{1}$ and mean $m_{k}^{\left(j\right)}$, respectively, and that all the channels are mutually independent. Then, the PDF of $\gamma_{1}$ is given by $f_{\gamma_{1}}\left(x\right)=e^{-x/m_{1}}/m_{1}$, and the PDF of $\gamma_{k}^{\left(j\right)}$ is given by $f_{\gamma_{k}^{\left(j\right)}}\left(x\right)=e^{-x/m_{k}^{\left(j\right)}}/m_{k}^{\left(j\right)}$. The CDF of Ω is then given by:

(36) $$\begin{array}{ccc} F_{\Omega }\left(x\right) & = & \prod_{j=1}^{K}\mathrm{Pr}\left\{\frac{\gamma_{5}^{\left(j\right)}}{\gamma_{4}^{\left(j\right)}\gamma_{6}^{\left(j\right)}}\leq x\right\}\\ & = & \prod_{j=1}^{K}\left\{1-\int_{0}^{\infty }\int_{0}^{\infty }F_{\gamma_{4}}\left(\frac{z}{xy}\right)f_{\gamma_{6}}\left(y\right)f_{\gamma_{5}}\left(z\right)dydx\right\}\\ & = & \prod_{j=1}^{K}\left\{1+\frac{\sigma^{\left(j\right)}}{x}e^{\frac{\sigma^{\left(j\right)}}{x}}\mathrm{Ei}\left(-\frac{\sigma^{\left(j\right)}}{x}\right)\right\}, \end{array}$$

where $\sigma^{\left(j\right)}=m_{5}^{\left(j\right)}/\left(m_{4}^{\left(j\right)}m_{6}^{\left(j\right)}\right)$ and $\mathrm{Ei}\left(x\right)=-\int_{-x}^{\infty }e^{-t}/tdt$.

**Lemma** **4.** $U_{AB}^{\max}\left(K\right)$ achieves full diversity gain in the sense that the log of utility outage probability decreases as an order of K when every $\sigma^{\left(j\right)}\to \infty$ and the channel gain $\gamma_{1}$ is known and fixed.

**Proof.**  Let us denote the fixed $\gamma_{1}$ by ${\bar{\gamma }}_{1}$, and we assume independent and identical $\gamma_{k}^{\left(j\right)}$’s (k=4,5,6), so that $\sigma =\sigma^{\left(j\right)}$ for all j. By using approximation $\mathrm{Ei}\left(-x\right)\overset{x\to \infty }{\approx }\frac{-e^{-x}}{x}\left(1-\frac{1}{x}\right)$ in (36), when σ→∞, ${\tilde{P}}_{\mathrm{out}}$ with given ${\bar{\gamma }}_{1}$ becomes:

(37) $$\begin{array}{ccc} {\tilde{P}}_{\mathrm{out}}\left({\bar{\gamma }}_{1}\right) & = & \mathrm{Pr}\left\{\Omega \leq \frac{\beta }{\left(\frac{\omega_{J}}{\mu \ln2}-1\right){\bar{\gamma }}_{1}}\right\}\\ & = & F_{\Omega }\left(\frac{\beta \mu \ln2}{\left(\omega_{J}-\mu \ln2\right){\bar{\gamma }}_{1}}\right)\\ & \approx & {\left(\frac{\beta \mu \ln2}{\left(\omega_{J}-\mu \ln2\right){\bar{\gamma }}_{1}}\right)}^{K}\sigma^{-K}. \end{array}$$

Thus,

(38) $$\begin{array}{c} \lim_{\sigma \to \infty }-\frac{\ln{\tilde{P}}_{\mathrm{out}}}{\ln\sigma }=K. \end{array}$$

 ☐

When Alice wants to keep its utility from dropping below a threshold μ, the increasing number of cooperative jammers probabilistically helps to reduce the probability of utility outage. Lemma 4 tells us how the number of jammers contributes to diminishing the outage probability. It tells us that when the channel between Jack and its receiver becomes relatively very good compared to the interfering links: Alice to $J_{R}$ and Jack to Bob, both of which are unwanted signal paths, the outage probability decreases log-linearly. This property also holds for jammers in $J_{N}$ even without assuming fixed $\gamma_{1}$. For the utility provided in Lemma 3, it is seen that only the ratio $m_{5}^{\left(j\right)}$/$m_{6}^{\left(j\right)}$ matters in achieving the full diversity gain for jammers in $J_{N}$.

### 4.3. Distributed Collaboration Protocol for Multiple Jammers

For a single jammer, Alice and Jack can share the whole channel knowledge by adequate feedback channels. However, if there are multiple (potential) jammers, it is hard for Alice and the jammers to have the wide-range channel information over the whole network. In this subsection, we assume that Alice has only the channel information on $\gamma_{1}$ and $\gamma_{2}$, and each jammer has its own channel information on $\gamma_{3}^{\left(j\right)}$, $\gamma_{4}^{\left(j\right)}$, $\gamma_{5}^{\left(j\right)}$ and $\gamma_{6}^{\left(j\right)}$.

Looking for the collaboration, Alice initially broadcasts λ with channel information $\gamma_{1}$, $\gamma_{2}$ and secrecy rate target $R_{AB}^{s}$ (and also $P_{A}$ if it is not known to the jammers in advance). Each jammer *j* that has some message to send and also seeks the collaboration opportunity then computes either $\alpha^{(j)*}\left(\lambda \right)$ or $P^{(j)*}\left(\lambda \right)$ according to (13) and (23), respectively. Of course, each jammer has prior knowledge of $\omega_{J}^{j}$ and $P_{\max}^{\left(j\right)}$. Each jammer sends either $\alpha^{(j)*}\left(\lambda \right)$ or $P^{(j)*}\left(\lambda \right)$ with $P_{\max}^{\left(j\right)}$, if needed, which could be already known to Alice by previous communications. Jammers send the power allocation as an agreement of the collaboration when the power allocation is positive. Alice receives the response from the jammers and tries to determine whether it will send a new price or stop sending the price and choose the best jammer with the feedbacks so far.

According to (13), $\alpha^{(j)*}\left(\lambda \right)$ is not dependent on λ if $\lambda \leq \lambda_{L}^{\left(j\right)}$. However, it decreases if $\lambda \geq \lambda_{L}^{\left(j\right)}$. Let us denote by $\lambda^{\left(i\right)}$ the *i*-th price successively sent by Alice looking for the collaboration. By using two successive designated prices $\lambda^{\left(i\right)}$ and $\lambda^{\left(i+1\right)}$ ($\lambda^{\left(i\right)}<\lambda^{\left(i+1\right)}$), Alice can have partial information on power allocation by jammer $j\in J_{C}$ from the responses such that if $\alpha^{(j)*}\left(\lambda^{\left(i\right)}\right)=\alpha^{(j)*}\left(\lambda^{\left(i+1\right)}\right)$, then $\alpha^{(j)*}\left(\lambda^{\left(i\right)}\right)=\alpha_{0}$ and $\lambda^{\left(i\right)}<\lambda^{\left(i+1\right)}\leq \lambda_{L}^{\left(j\right)}$. Getting $\alpha_{0}$ and a lower bound on $\lambda_{L}^{\left(j\right)}$ by the above reasoning, Alice can also have an upper bound on $\lambda_{L}^{\left(j\right)}$ by a response $\alpha^{(j)*}\left(\lambda^{\left(l\right)}\right)>\lambda^{\left(i+1\right)}$, which means $\lambda_{L}^{\left(j\right)}\leq \lambda^{\left(l\right)}$.

For jammer $j\in J_{N}$, its response on power allocation $P^{(j)*}\left(\lambda \right)$ will be one of $P_{L}^{\left(j\right)}$, $P_{U}^{\left(j\right)}$, $P_{\mathrm{opt}}^{\left(j\right)}$ and $P_{\max}^{\left(j\right)}$. When Alice increases λ, the response finally reaches either $P_{U}^{\left(j\right)}$ or $P_{\max}^{\left(j\right)}$: If $P_{U}^{\left(j\right)}\leq P_{\max}^{\left(j\right)}$, it eventually becomes $P_{U}^{\left(j\right)}$; Otherwise, $P_{\max}^{\left(j\right)}$. When Alice increases λ, regarding the sequence of responses, jammer $j\in J_{N}$ falls into one of three classes: the first type that responds with either fixed power $P_{U}^{\left(j\right)}$ or $P_{\max}^{\left(j\right)}$, the second type that increases its power from $P_{\mathrm{opt}}^{\left(j\right)}$ to $P_{U}^{\left(j\right)}$ and the third type that increases its power from $P_{\mathrm{opt}}^{\left(j\right)}$ to $P_{\max}^{\left(j\right)}$. Since Alice knows $P_{\max}^{\left(j\right)}$ from the feedback, it finally understands whether the jammer allocates $P_{U}^{\left(j\right)}$ or $P_{\max}^{\left(j\right)}$.

Motivated by the above findings, an interactive and iterative collaboration search procedure (ICSP) is provided in Table 2. It is primarily based on the bisectional search that guides Alice’s pricing trials to find $\lambda_{L}$ or ${\tilde{\lambda }}^{*}$. In this procedure, $\lambda_{\mathrm{small}}$ and $\lambda_{\mathrm{big}}$ represent a lower and an upper limit of the price in practice, respectively, which are set by Alice. ICSP tries to find the collaboration starting with the smallest possible price $\lambda_{\mathrm{small}}$, by which Alice can attract as many as friendly jammers possible. In ICSP, if $u_{j}\left|\lambda_{r,j}-\lambda_{l,j}\right|<\varepsilon$ for a predefined threshold ε, it is regarded that the price is sufficiently exploited for jammer *j*. If every potential jammer satisfies the condition, ICSP terminates with an individual pricing range of the jammers, and then, the most plausible jammer is selected for the collaboration. Otherwise, further improvement in the payment by jammer *j* is expected by $u_{j}\left(\frac{\lambda_{l,j}+\lambda_{r,j}}{2}\right)$ and the biggest jammer is selected for the next price-searching. Since $\frac{\lambda_{l,j}+\lambda_{r,j}}{2}$ replaces either $\lambda_{l,j}$ or $\lambda_{r,j}$ for some *j*, ICSP converges geometrically and terminates within $N\log_{2}\left|\left(\lambda_{\mathrm{big}}-\lambda_{\mathrm{small}}\right)/\varepsilon \right|$, where *N* represents the total number of potential jammers in $J_{C}\cup J_{N}$.

## 5. Numerical Examples

### 5.1. A Single Jammer

**Figure 2:**
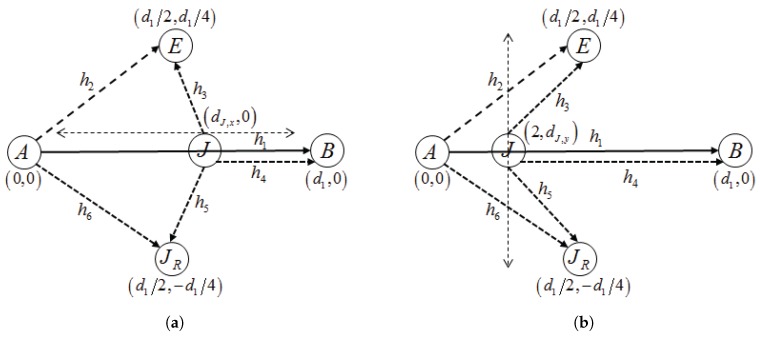
Network models used in the simulation: (**a**) the jammer moves horizontally; (**b**) the jammer moves vertically.

To illustrate the pricing mechanism proposed in this paper, we use the network configurations shown in Figure 2. The location of a communication node appears in a Euclidean (x,y) coordinate. Alice, Bob, Eve and $J_{R}$ are fixed at (0,0),$(d_{1},0),$$\left(d_{1}/2,d_{1}/4\right)$ and $\left(d_{1}/2,-d_{1}/4\right)$, respectively. Jack is assumed to move either from (0,0) to $\left(d_{1},0\right)$ along with the horizontal line (as shown in Figure 2a) or from (2,−1) to $\left(2,d_{1}/4\right)$ along with the vertical line (as shown in Figure 2b). The position of Jack is denoted by $\left(d_{J,x},0\right)\left(0\leq d_{J,x}\leq d_{1}\right)$ or $\left(2,d_{J,y}\right)\left(-1\leq d_{J,y}\leq d_{1}/4\right)$. We assume a geometric path loss law of $\gamma_{i}=a_{i}$/$d_{i}^{\nu }$ where $d_{i}$ is the distance of the respective link, ν is a path loss exponent and $a_{i}$ represents the effect of the other fading components. We also assume that $P_{A}$/$\sigma^{2}=P_{J}$/$\sigma^{2}=15$ dB, $\omega_{J}=1$ and $R_{AB}^{s}=0.1$.

**Figure 3:**
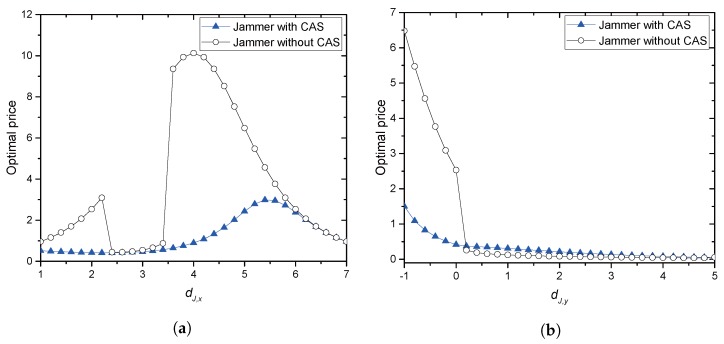
Optimal prices. (**a**) the jammer moves horizontally; (**b**) the jammer moves vertically.

Figure 3a,b illustrates the optimal prices obtained in this paper when Jack moves horizontally from Alice to Bob and vertically from (2,−1) to $\left(2,d_{1}/4\right)$, respectively. In Figure 3a, Jack in $J_{C}$ can be attracted to the collaboration with a relatively low price, for example less than 0.8940, until it reaches (4,0). Beyond this point, higher prices can be possible. Jack in $J_{N}$ allows a relatively high price compared to the price for $J_{C}$. However, in Figure 3b, Jack in $J_{N}$ mostly requires a low price for collaboration. When Jack moves along with the vertical line, the price goes down immediately after it is closer to Eve than to $J_{R}$, which means that the transmitting power contributes to jamming greatly, and hence, the price would be low.

**Figure 4:**
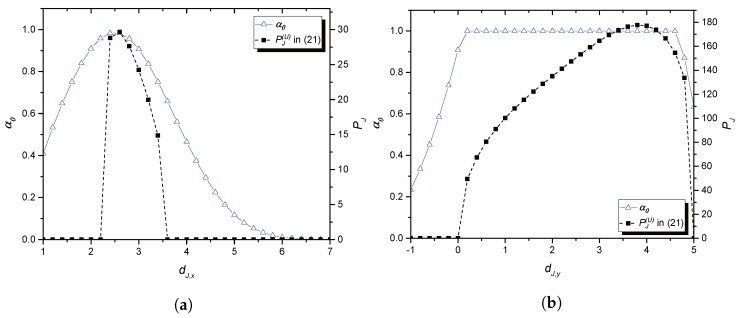
Power allocation for the collaboration: (**a**) the jammer moves horizontally; (**b**) the jammer moves vertically.

In Figure 4a,b, the resulting power allocation is provided for similar scenarios used (and thus, for the prices obtained) in drawing Figure 3a,b, respectively. In Figure 4a, it is seen that around position (2.6,0), the whole power is allocated to send Jack’s own signal for which the prices might be low. In Figure 4b, after Jack gets closer to Eve, the power allocated to send Jack’s own signal is increased with relatively low prices as described in Figure 3b. This is because the power is more efficiently used for jamming, and Jack enjoys low prices for sending its signal. However, if Jack goes too far from $J_{R}$ (for example, above (−2,4.6)), it is hard to enjoy the above favorable circumstances.

**Figure 5:**
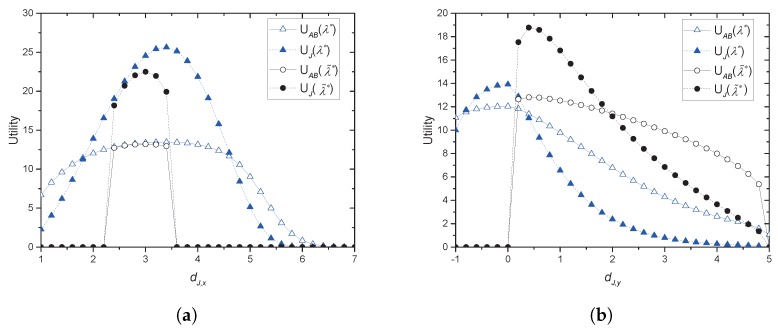
Utilities obtained by the collaboration: (**a**) the jammer moves horizontally; (**b**) the jammer moves vertically.

Figure 5a,b provides the utility values obtained by the collaboration, which is established by the prices obtained in Figure 3a,b, respectively. In both of the figures, it is seen that if Alice and jammers share CAS, then they have a significantly positive benefit at more positions than without CAS. For Figure 5a, significantly positive benefit for the collaboration without CAS is possible if it is between (2.4,0) and (3.4,0). With CAS, the range is widened to (1.0,0) and (6.4,0) in the tested scenario. In Figure 5b, the collaboration without CAS cannot achieve a positive benefit if Jack is closer to $J_{R}$ than to Eve. With CAS, there is no limitation on the collaboration. It is noted that the absolute values of the utilities are hardly compared since scale factors for the rate and the power are given arbitrarily for the simulation.

### 5.2. Multiple Jammers

To illustrate the behavior of optimal pricing, we randomly generate four jammers in $J_{C}$ (i.e., with CAS) and let each of them, at each time instant, move in the respective random direction of θ with fixed distance *r*. Between two successive time instants (i.e., two successive movements), the moving directions are designated to have a correlation of 0.8. The distance step distinguishes two classes of jammers: slow speed with r=0.1 and high speed with r=0.2. To test and compare, we use exactly the same trajectories for jammers in $J_{N}$ with those simulated for jammers in $J_{C}$. That is, along with a trajectory generated in the simulation, two jammers (one from $J_{C}$ the other from $J_{N}$) move together.

**Figure 6:**
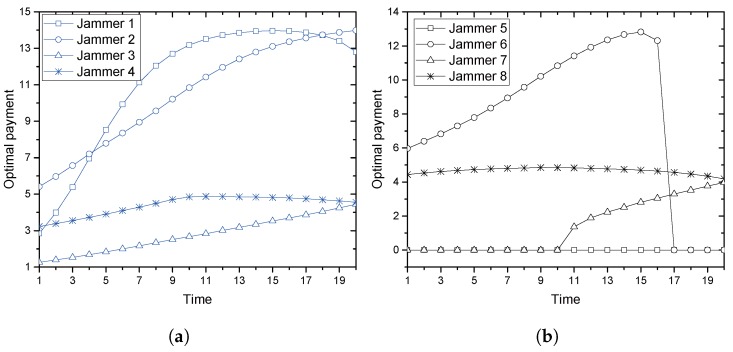
Comparison of payment to Alice from different jammers: (**a**) jammers from $J_{C}$; (**b**) jammers from $J_{N}$.

In Figure 6a,b, optimal $U_{AB}$ with each jammer at each time instant is illustrated. Jammers tested are indexed as $1,2,3,4\in J_{C}$ and $5,6,7,8\in J_{N}$, among which 1,2,5,6 are low-speed jammers and 3,4,7,8 are high-speed jammers. In Figure 6a, as time elapses from one to 20, optimal $U_{AB}$ payed by the jammers with CAS is plotted according to the movement. At the beginning, Jammer 2 is the best one that is willing to pay the most, and Alice would agree to the collaboration with Jammer 1. At Time Instant 5, Jammer 1 would pay more than Jammer 2 and be possibly selected as the collaboration partner until Time Instant 18. Jammers 3 and 4 have no chance in participating in the collaboration and to access the channel to send their own data. In Figure 6b, the payment by jammers without CAS, but with the same trajectories used, is plotted. In this group of jammers, Jammer 6 (on the same path with Jammer 2) enjoys the collaboration until Time 16, and Jammer 8 takes it from Time 17 to 20. Even on the same trajectories, the selection looks different. Jammer 1, the most frequently selected one if it has CAS, hardly has found a chance for collaboration without CAS.

**Figure 7:**
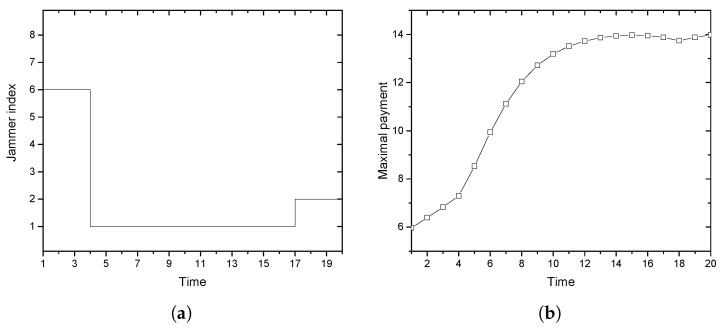
Multiple-jammer effects: (**a**) selected jammer by the greedy policy; (**b**) utility achieved by Alice with multiple jammers.

Figure 7a,b summarizes the selection result from Jammers 1 through 8 investigated in Figure 6a,b. In Figure 7a, the selected jammer is illustrated at each time instant. Until Time 3, Jammer 6 without CAS is selected. After Time 4, Jammer 1 and then Jammer 2 are selected, both of which have CAS. The resulting payment is illustrated in Figure 7b. It shows a 6.39%, 9.13% and 28.55% increase of the utility by Alice compared with a single jammer case of Jammers 1, 2 and 6, respectively.

**Figure 8:**
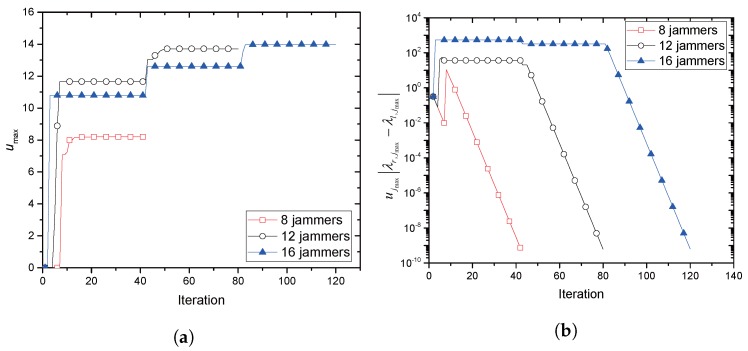
Convergence behavior of ICSP: **(a**) payment behavior; (**b**) convergence speed.

Figure 8a,b illustrates the convergence behavior of ICSP. ICSP with 8,12,16 potential jammers is tested, respectively. In Figure 8a, $u_{\max}$ (an updating variable that indicates the current greatest payment) is shown as iteration proceeds. $u_{\max}$, as expected, monotonically increases and almost approaches its maximum within around 15, 50 and 82 iterations with 8, 12 and 16 jammers, respectively. It is also seen that the increasing number of jammers provides a greater $u_{\max}$, which is increased by 24.53% and 37.67% with a 50% and 100% increase of jammers from eight, respectively. Figure 8b shows the maximum of convergence-testing metric $u_{j}\left|\lambda_{r,j}-\lambda_{l,j}\right|$ used in ICSP. If $\max\{u_{j}|\lambda_{r,j}-\lambda_{l,j}|\}<\varepsilon$, then ICSP terminates. It is seen that all three examples tested are terminated within around 23 iterations for $\varepsilon =10^{-4}$ and around 30 iterations for $\varepsilon =10^{-6}$ with eight jammers.

**Figure 9:**
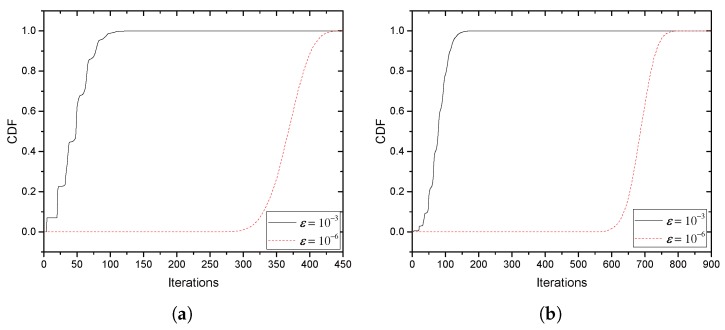
CDF of the number of iterations required for convergence of ICSP: (**a**) 20 jammers (**b**) 40 jammers.

Figure 9a,b provides the CDF of the number of iterations required to achieve target ε’s. Figure 9a,b tests the different numbers of jammers, 20 and 40, respectively, half of which are jammers with CAS and the other half without CAS. Instant collaborations from 10,000 random samples are used for each of the CDFs. With 20 jammers, more than 90% of tests are terminated within 78 and 401 iterations for $\varepsilon =10^{-3}$ and $10^{-6}$, respectively. Furthermore, with 40 jammers, more than 90% of tests are terminated within 113 and 734 iterations, respectively.

### 5.3. Comparative Result

In this subsection, we numerically compare the proposed method with an existing method that provides an optimal power allocation for friendly jammers [5] (referred to as OPF in the following) in terms of sum-rate (the sum of Alice’s secrecy rate and Jack’s data rate). The proposed method does not intend to maximize the sum-rate, but provides data rates that are determined by an optimal price. On the other hand, the method in [5] maximizes the secrecy rate between Alice and Bob, but does not allow Jack’s usage of the bandwidth. A direct comparison between the two methods is not fair. Thus, the purpose of this comparison is to provide an insight about how the proposed pricing allocates the limited radio resource between Alice and Jack compared with the existing optimal power allocation that just maximizes Alice’s data rate.

**Figure 10:**
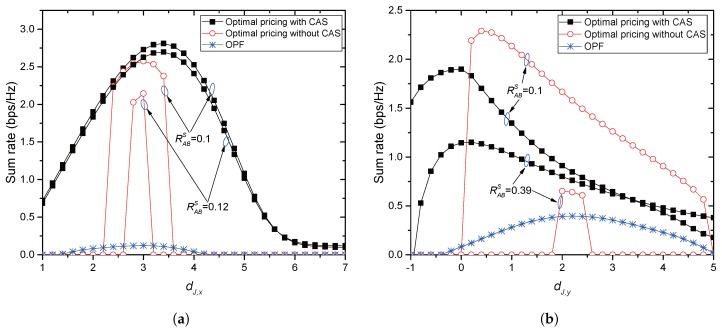
Comparison of sum-rates with a single jammer. (**a**) The jammer moves horizontally; (**b**) the jammer moves vertically.

Figure 10a,b compares the sum-rates provided by the optimal price given in this paper and OPF in [5]. For Figure 10a, Jack moves horizontally from Alice to Bob as used in Figure 3a. In this case, OPF provides its maximal rate of 0.12 at (3,0), from which the rate goes down since Jack approaches Bob. The proposed pricing achieves maximal sum rates of 2.81 and 2.57 at (3.4,0) and (3,0) with and without CAS, respectively, when the secrecy rate target $R_{AB}^{s}=0.1$. When $R_{AB}^{s}=0.12$, the proposed method achieves maximal sum rates of 2.69 and 2.14 at (3.4,0) and (3,0) with and without CAS, respectively. It is thus seen that the sum-rate achieved by the proposed method is significantly greater than the sum-rate by the optimal power allocation if the collaboration is established. The collaboration however can fail when large $R_{AB}^{s}$ is required especially without CAS. For Figure 10b, Jack moves vertically from (2,−1) to $\left(2,d_{1}/4\right)$ as used in Figure 3b. In this case, OPF provides its maximal rate of 0.39 at (2,2.2), to which the rate goes up since Jack gets close to Eve. The proposed pricing achieves maximal sum rates of 1.89 and 2.28 at (2,0) and (2,0.4) with and without CAS, respectively, when the secrecy rate target $R_{AB}^{s}=0.1$. When $R_{AB}^{s}=0.39$, the proposed method achieves maximal sum rates of 1.15 and 0.65 at (2,0.2) and (2,2) with and without CAS, respectively. In this figure, it is also seen that the proposed method can achieve the greater sum-rates if the collaboration is possible.

**Figure 11:**
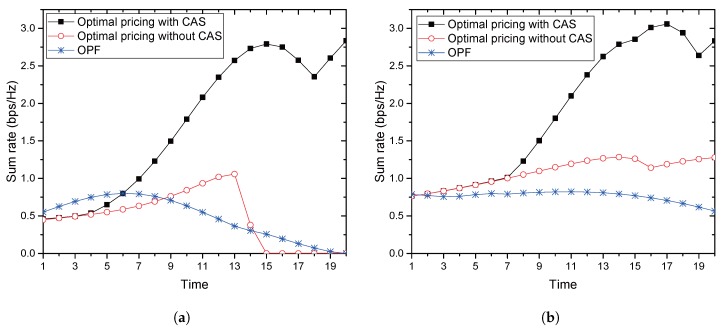
Comparison of sum-rates with multiple jammers. (**a**) When the number of jammers is four; (**b**) when the number of jammers is 10.

Figure 11a,b compares the sum-rates when multiple jammers exist, which randomly move according to the similar principle described in Subsection 5.2. In the simulation, we assume $R_{AB}^{s}=0.3$. In Figure 11a, the number of jammers is assumed to be four with and without CAS, respectively. OPF provides its maximal rate of 0.79 at the elapsing of Time 6, at which the proposed method achieves 0.79 and 0.58 with and without CAS, respectively. On the other hand, the proposed method achieves the maximum sum-rate of 2.8 and 1.05 at Times 20 and 13 with and without CAS, respectively. In Figure 11b, the number of jammers is assumed to be 10 with and without CAS, respectively. In this figure, OPF provides its maximal rate of 0.82 at the elapsing of Time 11, at which the proposed method achieves 2.09 and 1.19 with and without CAS, respectively. The proposed method achieves the maximum sum-rate of 3.05 and 1.28 at Times 17 and 14 with and without CAS, respectively. In comparison with Figure 10a,b and Figure 11a,b, it is seen that both of the methods enjoy a diversity gain from multiple friendly jammers. If the number of friendly jammers increases, the probability of collaboration also increases, and the proposed method could have a greater chance to achieve a greater sum-rate since it allows secondary usage of the radio resource by the jammers.

## 6. Conclusions

This paper has provided a pricing model to explain and encourage the proposed collaboration between Alice and friendly jammers. Optimal price and power allocation are analyzed and presented in closed-form. In wireless sensor networks, distributed sensor nodes often suffer from a lack of bandwidth and then can work as a jammer to share the bandwidth by the proposed collaboration. Greedy use of the bandwidth by jammers is limited by pricing the power used to send their own signal. For a multiple-jammer scenario, ICSP is provided for a practical implementation of the price-searching. The convergence is reached within 78 to 734 iterations depending on the number of participating jammers and the termination condition. To reduce the speed of finding a price, we limit the iterations by 10 and 20, respectively, motivated by Figure 8a and find that the average utility loss is only around 6% for both cases. Especially when the number of jammers is 40, the loss is 1.7% and 1.6%, respectively. This implies that the early termination does not cause significant loss in Alice’s utility and can be a promising method for massive sensor networks where a number of friendly jammers can exist. When considering 5 ms to be a typical period of radio scheduling in modern communication systems, a new good price can be searched within 50 to 100 ms for distributed multiple jammers. Finally, the channel assumption used in the simulation is somewhat limited, and we leave it for future study to apply more realistic fading environments such as, for example, correlated composite Nakagami-*m*/Gamma fading channels used in [14].

## Figures and Tables

**Table 1:** Notations.

Notation	Description
*A*	Source node (Alice)
*B*	Destination node (Bob)
$P_{A}$	Transmit power of Alice
*J*	Friendly jamming node (Jack)
*E*	Eavesdropper (Eve)
$J_{R}$	Receiver of jammers
$R_{AB}^{s}$	Secrecy rate target desired by Alice
λ	Price of power used to send jammer’s signal
$P_{J}$	Transmit power of a jammer
$x_{1}$	Complex symbol transmitted by Alice
$x_{2}$	Complex symbol transmitted by a jammer to $J_{R}$
$x_{J}$	Artificial jamming signals transmitted by a jammer
α	Power allocation factor between message signal $x_{2}$ and artificial jamming signal $x_{J}$
$y_{B}$	Received signal at Bob
$y_{J_{R}}$	Received signal at $J_{R}$
$y_{E}$	Received signal at Eve
$\eta_{B}$	Noise at Bob
$\eta_{J_{R}}$	Noise at $J_{R}$
$\eta_{E}$	Noise at Eve
$C_{AB}$	Channel capacity over link *A*-*B*
$C_{AE}$	Channel capacity over link *A*-*E*
$C_{AB}^{s}$	Secrecy rate on link *A*-*B*
$\alpha_{0}$	Maximally-allowable fraction of jammer’s power to its own signals while keeping CAC
$R_{J}$	Data rate of a jammer
$\omega_{J}$	Return per the data rate achieved by a jammer
$U_{J}$	Net revenue (utility) of a jammer
$U_{AB}$	Benefit of Alice by the collaboration
$J_{C}$	Set of jammers that have CAS
$J_{N}$	Set of jammers that have no CAS
$U_{AB}^{\max}\left(K\right)$	Alice’s utility with *K* cooperative jammers
$P_{\mathrm{out}}$	Utility outage probability
${\tilde{P}}_{\mathrm{out}}$	Asymptotic utility outage probability
Ei(·)	Exponential integral function

**Table 2:** Interactive collaboration search procedure (ICSP).

Step 0	(Initializing memories)
	Set $i=0,S_{s}=J_{C}\cup J_{N}$, $S_{a}=⌀$, $u_{\max}=0$, $j_{\max}=null,$
	and $\lambda_{l,j}=\lambda_{\mathrm{small}}$, $\lambda_{r,j}=\lambda_{\mathrm{big}}$, $u_{j}=\alpha_{0,j}=P_{0,j}=0$ for all $j\in S_{s}$.
	Set $\lambda^{\left(0\right)}=\lambda_{\mathrm{small}}$.
Step 1	(Broadcasting test price and receiving feedbacks)
	Try $\lambda^{\left(i\right)}$, and get $\alpha^{(j)*}\left(\lambda^{\left(i\right)}\right)$ from $j\in J_{C}$ and $P^{(j)*}\left(\lambda^{\left(i\right)}\right)$ from $j\in J_{N}$.
Step 2	(Updating jammers’ information)
	For $j\in J_{C}$,
	if i=0, then keep $\alpha_{0,j}=\alpha^{(j)*}\left(\lambda^{\left(i\right)}\right)$;
	if $u_{j}\leq \lambda^{\left(i\right)}\alpha^{(j)*}\left(\lambda^{\left(i\right)}\right)P_{\max}^{\left(j\right)}$, then update $u_{j}=\lambda^{\left(i\right)}\alpha^{(j)*}\left(\lambda^{\left(i\right)}\right)P_{\max}^{\left(j\right)}$
	and if $\alpha^{(j)*}\left(\lambda^{\left(i\right)}\right)=\alpha_{0,j}$ and $\lambda_{l,j}<\lambda^{\left(i\right)}$, then $\lambda_{l,j}=\lambda^{\left(i\right)}$; else if $\lambda_{r,j}>\lambda^{\left(i\right)}$, then $\lambda_{r,j}=\lambda^{\left(i\right)}$.
	if $u_{j}>\lambda^{\left(i\right)}\alpha^{(j)*}\left(\lambda^{\left(i\right)}\right)P_{\max}^{\left(j\right)}$ and $\alpha^{(j)*}\left(\lambda^{\left(i\right)}\right)\neq \alpha_{0,j}$ and $\lambda_{r,j}>\lambda^{\left(i\right)}$, then $\lambda_{r,j}=\lambda^{\left(i\right)}$.
	if $u_{j}>\lambda^{\left(i\right)}\alpha^{(j)*}\left(\lambda^{\left(i\right)}\right)P_{\max}^{\left(j\right)}$ and $\alpha^{(j)*}\left(\lambda^{\left(i\right)}\right)=\alpha_{0,j}$ and $\lambda_{l,j}<\lambda^{\left(i\right)}$, then $\lambda_{l,j}=\lambda^{\left(i\right)}$.
	For $j\in J_{N}$,
	if i=0, then keep $P_{0,j}=P^{(j)*}\left(\lambda^{\left(i\right)}\right)$;
	if $P_{0,j}\leq P^{(j)*}\left(\lambda^{\left(i\right)}\right)$ and $\lambda_{l,j}<\lambda^{\left(i\right)}$, then $\lambda_{l,j}=\lambda^{\left(i\right)}$.
	if $P_{0,j}\leq P^{(j)*}\left(\lambda^{\left(i\right)}\right)$ and $u_{j}\leq \lambda^{\left(i\right)}P^{(j)*}\left(\lambda^{\left(i\right)}\right)$, then $u_{j}=\lambda^{\left(i\right)}P^{(j)*}\left(\lambda^{\left(i\right)}\right)$.
	if $P_{0,j}>P^{(j)*}\left(\lambda^{\left(i\right)}\right)$ and $\lambda_{r,j}>\lambda^{\left(i\right)}$, then $\lambda_{r,j}=\lambda^{\left(i\right)}$.
Step 3	(Updating collaboration benefit and checking a termination condition)
	Find $u_{\mathrm{temp}}=\max\left\{u_{j},j\in J_{C}\cup J_{N}\right\}$
	and set $j_{\mathrm{temp}}$ as the corresponding jammer’s index such that $u_{j_{\mathrm{temp}}}=u_{\mathrm{temp}}$.
	If $u_{\mathrm{temp}}\geq u_{\max}$, then update $u_{\max}=u_{\mathrm{temp}}$ and $j_{\max}=j_{\mathrm{temp}}$.
	For $j\in S_{s}$, if $u_{j}\left|\lambda_{r,j}-\lambda_{l,j}\right|<\varepsilon$, then update $S_{s}=S_{s}-\left\{j\right\}$ and $S_{a}=S_{a}+\left\{j\right\}$.
	If $S_{s}=⌀$, then stop the procedure with selected jammer $j_{\max}$ and searched price $\lambda_{l,j_{\max}}$.
	Otherwise, i=i+1 and continue to Step 4.
Step 4	(Finding the most plausible jammer and updating searching price)
	Find $j^{*}$ such that $j^{*}=\arg\max\;\left\{u_{j}\left(\frac{\lambda_{l,j}+\lambda_{r,j}}{2}\right),j\in S_{s}\right\}$.
	Set $\lambda^{\left(i\right)}=\frac{\lambda_{l,j^{*}}+\lambda_{r,j^{*}}}{2}$ and repeat Step 1.

## Abbreviations

The following abbreviations are used in this manuscript:

CAS	Common artificial signal
Eves	Eavesdroppers
ICSP	Iterative collaboration search procedure

## References

[1] Wyner A.D. (1975). The Wire-Tap Channel. Bell Syst. Tech. J..

[2] Leung-Yan-Cheong S.K., Hellman M.E. (1978). The Gaussian Wiretap Channel. IEEE Trans. Inf. Theory.

[3] Zou Y., Zhu J., Wang X., Hanzo L. (2016). A Survey on Wireless Security: Technical Challenges, Recent Advances, and Future Trends. Proc. IEEE.

[4] Vilela J.P., Bloch M., Barros J., McLaughlin S.W. (2011). Wireless Secrecy Regions with Friendly Jamming. IEEE Trans. Inf. Forensics Secur..

[5] Cumanan K., Alexandropoulos G.C., Ding Z., Karagiannidis G.K. (2017). Secure Communications with Cooperative Jamming: Optimal Power Allocation and Secrecy Outage Analysis. IEEE Trans. Veh. Technol..

[6] Tekin E., Yener A. (2008). The General Gaussian Multiple-Access and Two-Way Wiretap Channels: Achievable Rates and Cooperative Jamming. IEEE Trans. Inf. Theory.

[7] Lai L., El Gamal H. (2008). The Relay-Eavesdropper Channel: Cooperation for Secrecy. IEEE Trans. Inf. Theory.

[8] Wang K., Yuan L., Miyazaki T., Zeng D., Guo S., Sun Y. (2017). Strategic Antieavesdropping Game for Physical Layer Security in Wireless Cooperative Networks. IEEE Trans. Veh. Technol..

[9] Li Z., Jing T., Ma L., Huo Y., Qian J. (2016). Worst-Case Cooperative Jamming for Secure Communications in CIoT Networks. Sensors.

[10] Liu Y., Li L., Alexandropoulos G.C., Pesavento M. (2017). Securing Relay Networks with Artificial Noise: An Error Performance-Based Approach. Entropy.

[11] Han Z., Marina N., Debbah M., Hjørungnes A. (2010). Physical Layer Security Game: Interaction between Source, Eavesdropper, and Friendly Jammer. EURASIP J. Wirel. Commun. Netw..

[12] Barros J., Rodrigues M.R.D. Secrecy Capacity of Wireless Channnels. Proceedings of the 2006 IEEE International Symposium on Information Theory.

[13] Kim I., Kim D. Pricing and Optimal Power Allocation in Collaborative Primary-Secondary Transmission using Superposition Coding. Proceedings of the 2010 IEEE Region 10 Conference (TENCON 2010).

[14] Alexandropoulos G.C., Peppas K.P. (2017). Secrecy Outage Analysis over Correlated Composite Nakagami-*m*/ Gamma Fading Channels. IEEE Commun. Lett..

